# Experimental and Numerical Investigation of Macroencapsulated Phase Change Materials for Thermal Energy Storage

**DOI:** 10.3390/ma17122804

**Published:** 2024-06-08

**Authors:** Busra Arslan, Mustafa Ilbas

**Affiliations:** 1 Department of Energy Systems Engineering, Faculty of Technology, Gazi University, Ankara 06570, Turkey; ilbas@gazi.edu.tr; 2 Department of Metallurgy and Material Engineering, Faculty of Engineering and Natural Sciences, Iskenderun Technical University, Hatay 31200, Turkey; 3 Graduate School of Natural and Applied Science, Gazi University, Ankara 06570, Turkey

**Keywords:** phase change material, paraffin, heat exchanger, aluminum encapsulation, heat storage

## Abstract

Among the different types of phase change materials, paraffin is known to be the most widely used type due to its advantages. However, paraffin’s low thermal conductivity, its limited operating temperature range, and leakage and stabilization problems are the main barriers to its use in applications. In this research, a thermal energy storage unit (TESU) was designed using a cylindrical macroencapsulation technique to minimize these problems. Experimental and numerical analyses of the storage unit using a tubular heat exchanger were carried out. The Ansys 18.2-Fluent software was used for the numerical analysis. Two types of paraffins with different thermophysical properties were used in the TESU, including both encapsulated and non-encapsulated forms, and their thermal energy storage performances were compared. The influence of the heat transfer fluid (HTF) inlet conditions on the charging performance (melting) was investigated. The findings demonstrated that the heat transfer rate is highly influenced by the HTF intake temperature. When the effect of paraffin encapsulation on heat transfer was examined, a significant decrease in the total melting time was observed as the heat transfer surface and thermal conductivity increased. Therefore, the energy stored simultaneously increased by 60.5% with the encapsulation of paraffin-1 (melting temperature range of 52.9–60.4 °C) and by 50.7% with the encapsulation of paraffin-2 (melting temperature range of 32.2–46.1 °C), thus increasing the charging rate.

## 1. Introduction

Phase change materials (PCMs) are preferred in thermal energy storage systems due to their excellent energy storage properties [1,2]. In particular, using PCMs in implementations such as solar power systems [3,4] and waste heat recovery systems [5,6] plays an important role in improving the energy storage efficiency of these systems. Recently, scientists have been focusing on such thermal storage systems by investigating the properties of PCMs, such as a high storage capacity for low-temperature applications. Various strategies have been attempted to improve the efficiency of these heat recovery systems, including encapsulation [7,8], the use of fins [9], and nanoparticle incorporation [10]. Thus, heat exchanger configurations containing PCMs have been investigated, both numerically and experimentally, as latent heat thermal storage systems [11].

In order to comprehend the function of buoyancy-induced convection during the restricted melting of PCMs in a tube and shell heat exchanger, Hosseini et al. [12] conducted a combined computational and experimental investigation. A number of tests were carried out to determine how raising the HTF’s intake temperature affects the PCM’s melting process. The simulation of the phase change phenomena was based on an iterative, finite-volume, numerical approach using a single-domain enthalpy formulation. The melting front occurred at different times at sites near the HTF pipe and moved toward the shell at varied speeds, according to the experimental data. According to the computational findings, raising the intake water temperature to 80 °C reduced the overall melting time by 37%. A heat exchanger was built, put together, and run by Cano et al. [13] in order to assess the performance of several PCMs. A thermal storage system, the heat exchanger unit, was employed to recover the remaining energy from the hydrogen cycle. After that, it may be applied to the building of air conditioning systems. Due to their advantageous thermal characteristics, four PCMs (microencapsulated PCM35, rubitherm RT55, rubitherm RT48, and rubitherm RT28) were chosen. The PCMs were securely kept inside the shell, while water served as the HTF. Rubitherm RT48 provided the highest thermal performance out of all the PCMs examined, since it was able to store the most energy. It was also determined how the HTF flow rate affected the shell and tube heat exchanger’s thermal efficiency. High heat transfer values were the result of low HTF flow rates. Ultimately, microencapsulated PCM35 was used to evaluate two operational modes: countercurrent PCM flow and waterproof PCM flow. The best experimental system for energy transmission was found to be the PCM countercurrent flow system, which achieved heat accumulation values that were almost 71% greater than those seen in the waterproof mode.

In-depth experimental research was conducted by Sodhi et al. [14] to examine the performance attributes of a latent heat storage (LHS) system. The HTF used was air in the unique experimental setup that was devised, with a maximum temperature of 400 °C. A multi-tube heat exchanger’s shell side was filled with sodium nitrate, which was utilized as a phase transition material. By altering the flow rate and intake temperature, the performance metrics, such as the output power, charge/discharge time, and discharged/stored energy, were calculated. During the charging process, heat transfer is generally caused by natural convection, according to the axial and radial temperature distributions, whereas conduction heat transfer in the axial direction is primarily responsible for the discharge. A maximum temperature of 365 °C for the PCM was reached, resulting in energy storage of approximately 19.5 MJ. The LHS medium has a high energy storage density and a low storage cost when compared to sensible heat storage media made of concrete and cast steel that operate under comparable testing settings. Nevertheless, different load requirements can be satisfied by combining sensible heat storage and the LHS medium. Rahimi et al.’s study [11] examined the solidification and melting of a PCM in a fin and tube heat exchanger. R35 material, which is intended to store water’s energy as the HTF flowing within the tubes, filled the shell side, which included the area around the tubes and the gaps between the fins. The flow rate, input temperature, and geometrical factors (fin spacing) were examined in relation to their effects on the PCM’s charging and discharging processes using the experimental setup. A continuous spiral tube that formed a heat transfer surface was located on the shell side, which was a rectangular cube. The findings demonstrated that, independently of the flow regime, the use of fins raised the average fin temperature. For all the regimes, the reduction in fin spacing had no discernible impact on this parameter. The melting time dropped more noticeably when the input temperature rose from 50 to 60 °C than when it rose from 60 to 70 °C.

Kanimozhi et al. [15] constructed an experimental design and evaluated the effectiveness of a TES system for enhancing heat transfer by utilizing honey and paraffin waxes. The tests were conducted within a TES cylindrical tank, manufactured and embedded with copper tubes that held liquid paraffin waxes. The purpose of this non-isothermal system’s construction was to increase the pace at which water from the solar tank unit heated the waxes within the thermal chamber. By using this technology, thermal energy is saved and a better option than a traditional storage tank is offered. After conducting and analyzing the trials, data were gathered during the PCM charging and discharging operations and the performance was evaluated. Consequently, it was discovered that, throughout the charging and discharging phases of this storage system, the honey and paraffin waxes contributed more than 40% to the heat recovery. The test findings of a small-scale hybrid sensible/latent heat storage system with water and macroencapsulated PCMs added were given by Frazzica et al. [16]. In the laboratory, two distinct PCMs, a commercially available mixture of hydrate and paraffin salts, were macroencapsulated and put into the testing tank. The researchers investigated several volume ratios between the PCMs and water. Different types of home hot water were simulated in order to conduct the testing. Even for the small percentages of the PCM utilized, the resultant data demonstrated a considerable improvement in the heat storage capacity per unit volume (1.3 dm^3^). Macroencapsulated paraffin was employed by Hawlader et al. [17] as the core material for thermal energy storage in a packed bed. Studies using simulations and experiments were conducted to assess the characteristics of encapsulated PCMs. The simulation investigation was conducted using the Fluent 18.2 software. The ability of the encapsulated PCM to establish hydrogen bonds with water, its hydrophilicity, and its energy storage capacity were assessed, together with its encapsulation rate. A higher paraffin encapsulation rate was shown to be correlated with a higher coating/paraffin ratio. The paraffin-to-coating ratio is the primary determinant of the hydrophilicity of encapsulated paraffin. The hydrophilicity of the product decreases with an increasing ratio. Due to varying paraffin/coating ratios, the encapsulated paraffin demonstrated substantial energy storage and release capabilities (20–90 J/g) during phase changes. According to the study, the encapsulated paraffin maintained both its geometric profile and its ability to store energy even after 1000 heat cycles.

A different potential use for a macroencapsulated LHS device was examined by Kannan et al. [18]. Their objective was to enhance the yield of a hemispherical solar still by utilizing paraffin wax as particulate matter (PCM), which was enclosed in used aluminum cans. By contrasting the performance of the solar still with the PCM and the solar still without the PCM, an analysis was also conducted to determine the ideal mass of the PCM. Their findings demonstrated that the PCM encapsulated in aluminum cans added 92.80% more clean water to the solar still pool than did the naked solar still without any PCM. By increasing water evaporation, the aluminum cans containing paraffin wax efficiently harnessed heat to raise the production of drinking water. Aluminum cans with a good conductivity and the maximum PCM mass showed excellent potential as a cheaper encapsulation material. A significant increase in the efficiency of the LHS system and the efficiency of the spherical solar collector system was achieved with the macroencapsulated PCM. A similar study was conducted by Thakur et al. [19] to purify brackish water using a tubular solar still. Consequently, they employed a PCM in the form of closed mild steel tubes packed with paraffin wax and carbon nanoparticles. In order to increase water production, three separate tests were carried out: one using a normal solar still without any PCM, and the other two using a PCM that was doped with nanoparticles in the amount of 0.3 percent by weight and a PCM that was merely encapsulated. There was a 40.59% increase in the thermal conductivity when comparing the PCM-only system to the system with 0.3 wt% nanoparticles. In addition, the nanoparticles changed the phase change temperatures with minimal deviation from the ground state. Compared to the conventional system without any PCM addition, the encapsulated PCM and the PCM doped with 0.3% nanoparticles increased water production by 42.4% and 87.82%, respectively. It was also found that the PCM and the PCM doped with 0.3 wt% nanoparticles resulted in 59.26% and 109.94% higher energy efficiencies than the conventional case. It was determined that the thermally well-performing, encapsulated, nanoparticle-doped PCM greatly enhanced water production and could be widely used in various distiller designs to achieve an improved thermal performance.

Zukowski et al. [20] determined the performance characteristics of a TES unit based on macroencapsulated paraffin wax. He used RII-56 (paraffin wax) as a storage medium. His study presented experimental results, including the tested unit’s charge, discharge, and pressure drop characteristics. The heat storage capacity of the unit varied between 240 and 262 kJ/kg as the PCM temperature changed from 49 °C to 57 °C. The research data showed that PCM RII-56 can be successfully used in heat storage applications. By adding a PCM to the layers of walls and ceilings, Hasan et al. [21] carried out an experimental investigation on the material’s potential for use as thermal insulation. The walls and ceiling included an aluminum frame that encased the PCM. Through experiments, the impact of the PCM and its function in enhancing the thermal comfort and performance were examined. Two-room models that included a standard room for comparison and an experimental room for testing were built. The paraffin wax PCM type utilized in the studies had a melting point of 44 °C. Several examples were examined based on the PCM’s thickness and orientation (ceiling, east wall, west wall, north wall, and south wall). According to the results, using a PCM as an insulating material lowers the zone’s internal temperature and cooling load, which lowers the power usage.

The literature evaluation summarized above discusses the use of PCMs with heat exchangers and different types of heat exchanger designs to increase the heat transfer between the PCM and the HTF. It was concluded that paraffin wax is the most valuable type among the different PCM types examined in the literature. However, its low thermal conductivity and limited operating temperature range, along with leakage and stabilization problems, are the main barriers to the use of paraffin wax in applications. Therefore, various studies have used encapsulation, metal fins, the addition of porous materials with a high thermal conductivity, the addition of nanoparticles, etc., to improve paraffin’s thermal performance. Among these, encapsulation has proven to be one of the most promising methods for increasing heat transfer. Therefore, in this study, to minimize the disadvantages of paraffin, a thermal energy storage unit was designed using aluminum alloy macrocapsules with cylindrical shells, which had a high thermal conductivity and mechanical strength. The melting temperature range for paraffin produced in Turkey is between 45 and 90 °C. In this study carried out within domestic facilities, a paraffin with a lower melting point (melting temperature range of 32.2–46.1 °C), made by a commercial company in a laboratory environment, was experimentally and numerically tested. The thermal energy storage behavior of this new paraffin was investigated.

## 2. Experimental Setup and Studies

**Figure 1:**
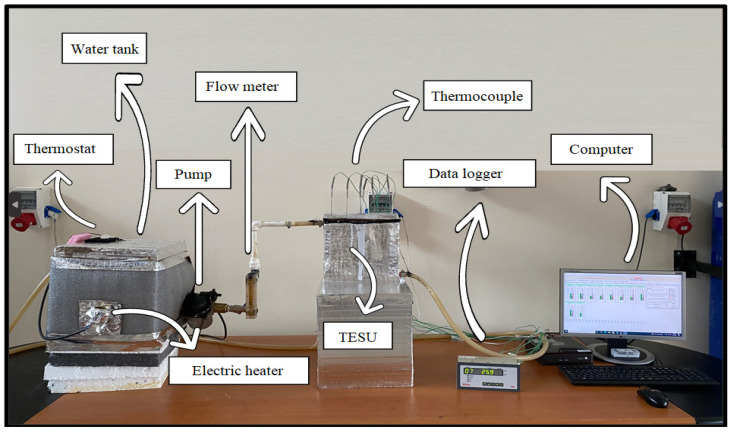
A general overview of the experimental setup.

A general overview of the experimental setup is given in Figure 1. The experimental setup comprised a 20 lt stainless steel insulated water tank, a 2000 W electric water heater, a thermostat, a 3-stage 65 W pump, a flow meter with a measurement range of 2–18 lt/min, a 23 cm × 23 cm × 3 cm copper TESU, and a copper heat exchanger with a diameter of 10 mm contacting one surface of the TESU. The experimental setup also consisted of K-type thermocouples that measured the temperature from different points of the TESU and also from the inlet and outlet points of the water; a 16-channel data logger that allowed the measurement results to be obtained; and a computer where the received data were stored.

**Figure 2:**
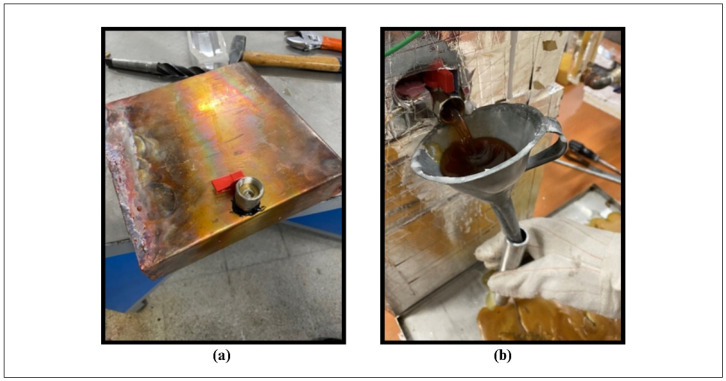
(**a**) Drain tap mounted on TESU; (**b**) draining process of melted PCM.

To improve the thermal performance, a 1 mm thick copper sheet with a high thermal conductivity coefficient was used in the design of the TESU and the heat exchanger. The copper heat exchanger had a diameter of 10 mm and a thickness of 1 mm to allow the HTF to pass through. The HTF was water heated by an electric heater. The water tank, heat exchanger, and TESU were insulated to avoid thermal losses. Figure 2a shows the drain cock installed in the TESU, and Figure 2b shows the draining process of the melting liquid PCM. The thermocouples (T10, water inlet; T11, water outlet) placed inside the circulation pipes to measure the water inlet temperature and outlet temperature into the TESU were coated with water-resistant epoxy resin to prevent leaks.

**Figure 3:**
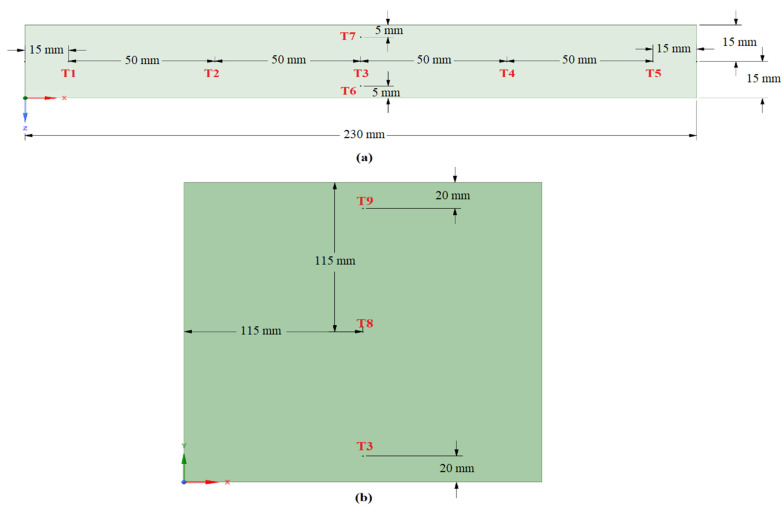
Locations of the thermocouples in the PCM along (**a**) the XZ-axis and (**b**) the XY-axis.

Before starting the experiments, the TESU was filled with a molten PCM in a controlled manner, and before freezing, the thermocouples were placed at the locations identified in Figure 3. The thermocouples along the XZ-axis were placed in plane 5, described in the numerical analysis section, to obtain a reliable temperature profile and to avoid contact with the TESU surface. The thermocouples along the XY-axis were placed to study the temperature variation in the radial direction.

After the thermocouple placement was completed, the PCM was allowed to cool in ambient air. The PCM was in thermal equilibrium when the experiment began (13–17 °C). After that, the HTF was heated to the desired temperature. When this temperature was reached, the pump was started. The experiment was carried out until the PCM peak temperature was reached. The experiments were conducted by considering the three different HTF flow rates of 0.0333 lt/s, 0.0666 lt/s, and 0.1 lt/s, and the three different temperatures of 60 °C, 70 °C, and 80 °C. During the charging process, the temperature was measured at specific points in the TESU with thermocouples at one-minute intervals and recorded with a data logger. The TESU was covered with a modular insulated copper cover.

To interpret the thermal performance of different PCMs, two paraffins with different physical properties (one white and the other yellow) were placed in the TESU, and their temperatures were measured at various times. Paraffin is a saturated n-alkane aliphatic hydrocarbon. These are represented by a general formula (CH_3_–(CH_2_)n–CH_3_), where n is the number of carbon atoms. The melting point increases with the number of carbon atoms in the backbone chain. Pure paraffin requires a high level of refinement and is expensive. Instead, a cheaper commercial paraffin wax, a by-product of petroleum refining, was used as the PCM in the thermal energy storage system. Paraffin-1 and paraffin-2 are basically paraffin mixtures with different carbon atomic numbers. Paraffin-1 is a mixture of paraffin with C_26_-C_30_ carbon atoms and contains 3–5% oil. Paraffin-2 is a mixture of paraffin with C_15_-C_26_ carbon atoms and contains 10–15% oil. The paraffins were supplied by a commercial company. The properties of the paraffin and copper in the TESU are given in Table 1.

The PCMs P1 and P2 were used directly in the rectangular volume of the TESU and placed in macro-sized aluminum tubes with a diameter of 2.5 cm. Encapsulation refers to the coating of solid, liquid, or gaseous substances with a shell layer of a macro, micro, or nano size. The purpose of encapsulating PCMs is to reduce the exposure of the inner material to external influences, keep the liquid material at a specific volume during the phase change process in the solid shell, and increase the heat transfer surface. The temperatures inside the encapsulated PCMs were measured with thermocouples throughout the experiment.

**Figure 4:**
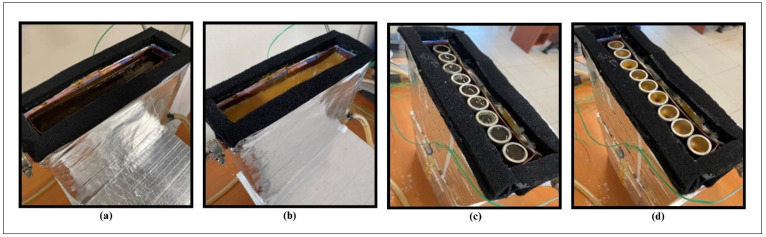
Phase change processes in TESU: (**a**) melting of P2; (**b**) solidification of P2; (**c**) melting of EP2; and (**d**) solidification of EP2.

The capsules were heated with the HTF to a temperature over the melting point of the PCM (up to the peak temperature) and then cooled to under the melting point to solidify the PCM. Figure 4 shows the state of P2 and encapsulated paraffin-2 (EP2) in the TESU at the end of the melting and solidification processes.

## 3. Numerical Analysis

### 3.1. Governing Equations

The enthalpy approach was used in this study because it provided a few benefits. The enthalpy method is used to solve phase change problems involving both conduction and convection. The flexibility of the enthalpy approach allows it to be applied when the phase change takes place at a single temperature ($T_{m}$) or throughout a range of temperatures ($T_{m}-\Delta T$) and ($T_{m}+\Delta T$). This flexibility is the primary benefit of the method. Since a fixed mesh structure is used in this method, it provides computational convenience [22]. In the enthalpy method, the sensible heat and latent heat terms are combined in the total enthalpy term. The solid–liquid interface position cannot be precisely determined using the enthalpy approach, since the governing equation is the same for both the liquid and solid phases. A mushy zone that divides the two phases indicates the interface [23]. For constant thermophysical parameters, energy conservation is represented by the temperature and the total volumetric enthalpy.

(1) $$\frac{\partial \rho H}{\partial t}+\nabla .(\rho vH)=\nabla .(k\nabla T)+S$$

where H is the sum of the sensible and latent heat found in Equations (2) and (3), so H is the total volumetric enthalpy, and the sensible heat at $T_{ref}$ is $h_{ref}$:

(2) H=h+fL

(3) $$h=h_{ref}\int_{T_{ref}}^{T}C_{p}dT$$

where *f*, which is defined in Equation (4) and represents the liquid fraction, indicates the percentage of the cell volume in liquid form. A zone with a liquid ratio between 0 and 1 is known as a mushy zone [23].

(4) $$f=\left\{\begin{array}{cc} 0 & ise\;T<T_{solid}\\ \frac{T-T_{solid}}{T_{liquid}-T_{solid}} & ise\;T_{solid}<T<T_{liquid}\\ 1 & ise\;T>T_{liquid} \end{array}\right.$$

By applying Equations (2)–(4), the energy equation can be written as follows:

(5) $$\frac{\partial \rho h}{\partial t}+\nabla .\left(\rho vh\right)=\nabla .\left(k\nabla T\right)-\frac{\partial \rho fL}{\partial t}-\nabla .\left(\rho vfL\right)+S$$

If we take into account the effect of natural convection due to the melt density change in the material, the momentum equation can be written as follows:

(6) $$\frac{\partial \rho v}{\partial t}+\nabla .(\rho vv)=-∆P+\nabla .(\mu \nabla v)+\rho g+\frac{{\left(1-f\right)}^{2}}{f^{3}+\varepsilon }vA_{mush}$$

Here, v is the velocity and $A_{mush}$ is a constant that describes how steeply the velocity is reduced to zero when the material solidifies, reflecting the morphology of the mushy zone. The constant ε is a small number to avoid divergence to zero.

Because it converges more quickly than other temperature-dependent models, the Boussinesq approximation was employed in the numerical analysis. This approximation is appropriate if the density variation is minimal. The model was based on the temperature ($T_{0}$), reference density ($\rho_{0}$), and volumetric expansion coefficient (β), and it assumed that the fluid density was constant throughout the momentum equation, with the exception of the force term. The momentum equation can be expressed as follows [23]:

(7) $$\frac{\partial \rho_{0}v}{\partial t}+\nabla .(\rho_{0}vv)=-∆P+\nabla .(\mu \nabla v)+(\rho -\rho_{0})g+\frac{{\left(1-f\right)}^{2}}{f^{3}+\varepsilon }vA_{mush}$$

(8) $$\left(\rho -\rho_{0}\right)g=-\rho_{0}\beta \left(T-T_{0}\right)$$

The continuity equation is given in [22]:

(9) $$\frac{\partial \rho }{\partial t}+\nabla .(\rho v)=0$$

Equations (5) and (9) are solved in the conduction-only model. Equations (5) and (7)–(9) may be solved when the combined conduction/convection heat transfer process is considered. The enthalpy balance calculates the liquid fraction in each domain cell during each iteration.

### 3.2. Numerical Procedure

**Figure 5:**
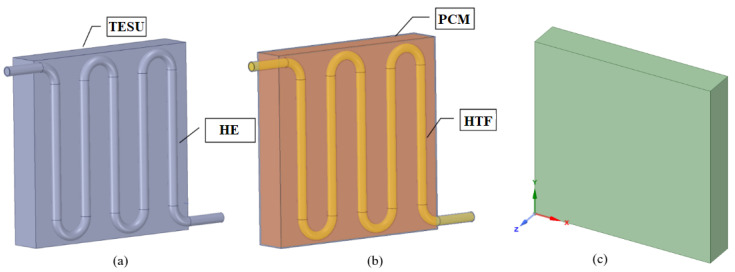
(**a**) Thermal energy storage unit and heat exchanger; (**b**) phase change material and heat transfer fluid; and (**c**) numerical model of the numerically analyzed thermal energy storage unit.

The numerical studies were performed with the Ansys 18.2 software, and the melting processes for laminar flow were simulated using the enthalpy-porosity approach and the Navier–Stokes equations. The fluid dynamics equations were solved simultaneously in all the computational domains with the COUPLED algorithm in Fluent’s solver setting. While the PRESTO (pressure-staggering option) method was preferred for pressure, the SECOND ORDER UPWIND approach was used for the momentum and energy equations. These methods were selected to analyze the natural convection of fluids precisely and to obtain realistic results. The TESU, heat exchanger (HE), HTF, and PCM were drawn with the SpaceClaim-18.2 three-dimensional modeling application in the Geometry part of Ansys (Figure 5a,b). To facilitate the solution, the heat exchanger and the HTF were removed from the geometric model, and the average heat flux from the HTF to the PCM during the total melting time was defined in the program. Figure 5c shows the three-dimensional drawing of the numerically analyzed TESU created in SpaceClaim.

**Figure 6:**
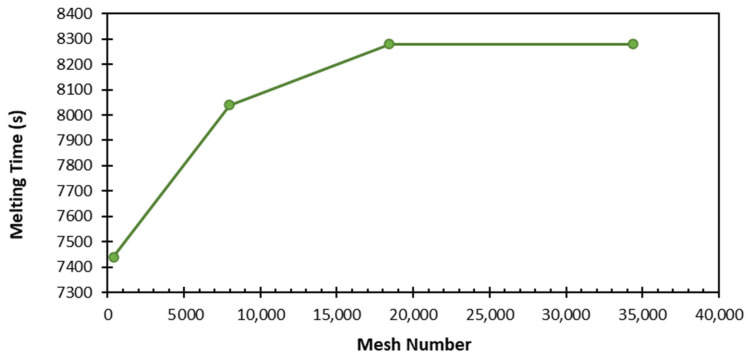
Variation in total PCM melting time depends on number of meshes.

**Figure 7:**
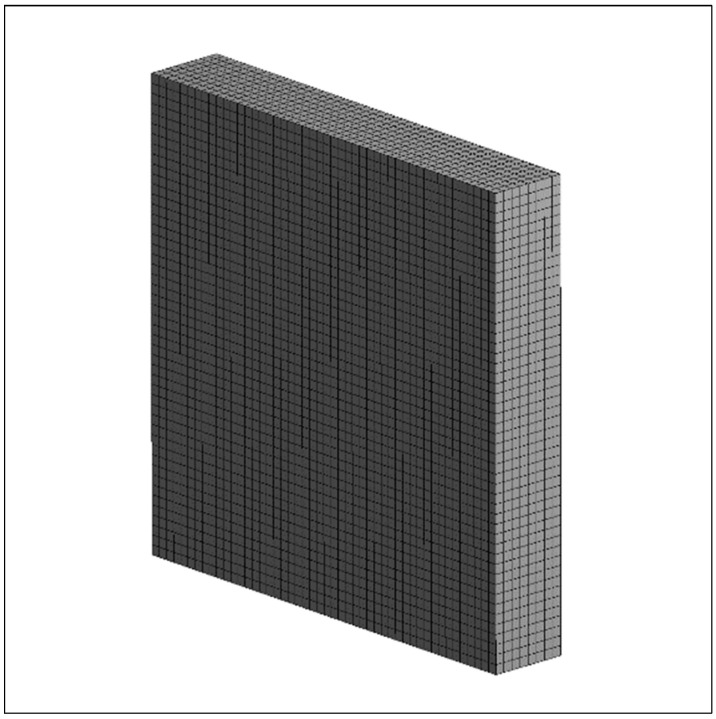
Mesh structure of the thermal energy storage unit.

In the three-dimensional time-dependent study, four different element numbers—392; 7975; 18,432; and 34,398—and three different time steps—0.5, 0.1, and 0.2—were used. As a result of these studies, the total melting time of the PCM did not change significantly with an increasing mesh number, and no change was observed after 18,432 elements (Figure 6). Considering the optimal value and time, the time step of 0.1 s and 18,432 elements were adequate for the study. The numerical analysis found that the minimum–average–maximum element quality and orthogonal quality values were above 0.99, and the skewness value was below 0.1. This supports the conclusion that the model had a high-quality mesh structure. Figure 7 shows the mesh structure of the TESU.

**Figure 8:**
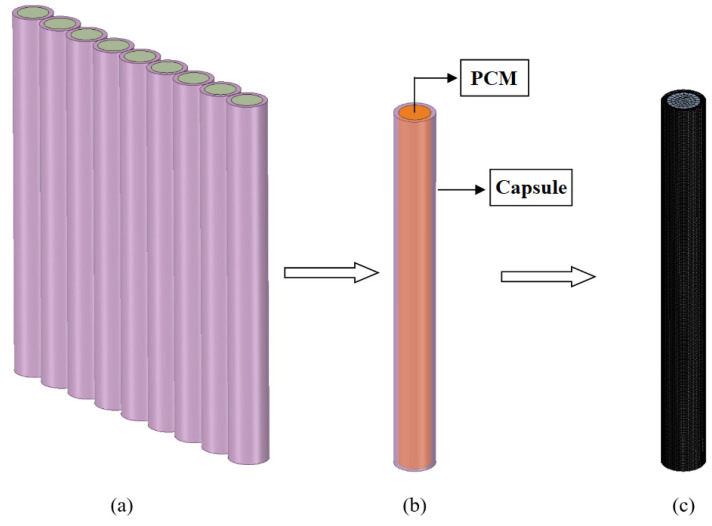
Drawings of PCM capsules: (**a**) PCM with nine capsules; (**b**) numerical model of the encapsulated PCM; and (**c**) mesh structure of the encapsulated PCM.

Numerical modeling was performed both for the PCM placed directly inside the TESU and for the encapsulated PCM. Nine macro-sized capsules were used, with an inner diameter of 1.9 cm, an outer diameter of 2.5 cm, a wall thickness of 0.3 cm, and a length of 23 cm, and the PCMs inside the capsules were drawn with the SpaceClaim three-dimensional modeling application in the Geometry section of Ansys (Figure 8a). The other capsules were removed from the model and a PCM with a single capsule was analyzed to facilitate the solution. A 1000-series aluminum alloy was used as the capsule material. This is a type of aluminum alloy comprising 99% pure aluminum and small amounts of other elements. The heat conduction coefficient of the aluminum alloy was 218 W/mK. The average heat flux from the HTF to the capsule over the total melting time in the experimental study was defined on the capsule surface. Figure 8b shows a three-dimensional drawing of the numerically analyzed PCM with the capsule, and Figure 8c shows the mesh structure.

**Figure 9:**
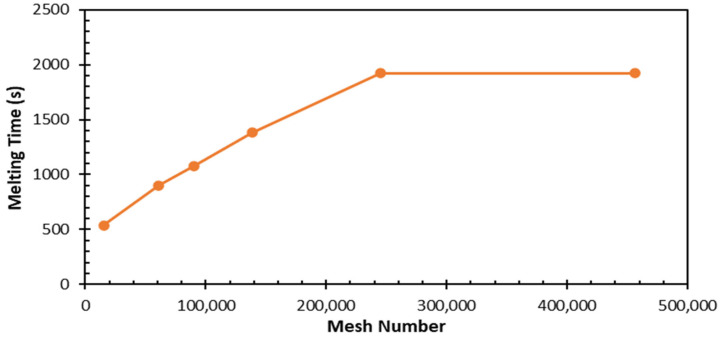
Variation in total encapsulated PCM melting time depends on number of meshes.

In the three-dimensional time-dependent study investigating the encapsulated PCM, mesh independence was performed, and six different element numbers—15,556; 61,086; 90,276; 138,369; 244,836; and 456,298—were studied. As a result of the studies, it was observed that the total melting time of the PCM increased with an increase in the mesh number, and no changes were observed after 244,836 elements (Figure 9). Considering the optimal value and time, 244,836 elements was adequate for this study.

## 4. Results and Discussion

### 4.1. Model Validation

**Figure 10:**
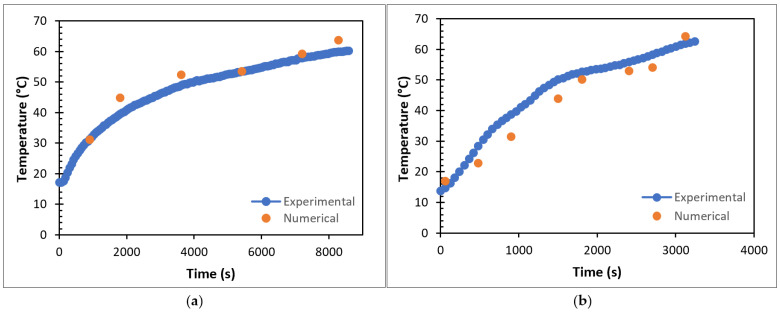
Comparison of numerical analysis results and experimental results of (**a**) P1 and (**b**) EP1.

The model accuracy was tested by comparing the results of the experimental studies and the numerical analysis results. This test was performed by comparing the values obtained for the total melting time of P1 and EP1 and the time-dependent variation in T3 at a reference temperature of 80 °C and a reference flow rate of 0.0333 lt/s. The comparison of the numerical analysis results and experimental results for P1 in the TESU is shown in Figure 10a, while the comparison of the results for EP1 is shown in Figure 10b.

As a result of the comparison, it was observed that the experimental results and numerical modeling were compatible with each other. As seen in Figure 10a, the total melting time of P1 was 8280 s in the numerical analysis results, while this value was found to be 8580 s in the experimental results. Similar results were found when the numerical and experimental results of encapsulated paraffin were compared. As seen in Figure 10b, the total melting time of EP1 was 3120 s in the numerical analysis results, while this value was 3240 s in the experimental results. In addition to the total melting time, the time dependence of the temperature at T3 for both P1 and EP1 was analyzed for both cases.

### 4.2. Numerical Analysis Results

**Figure 11:**
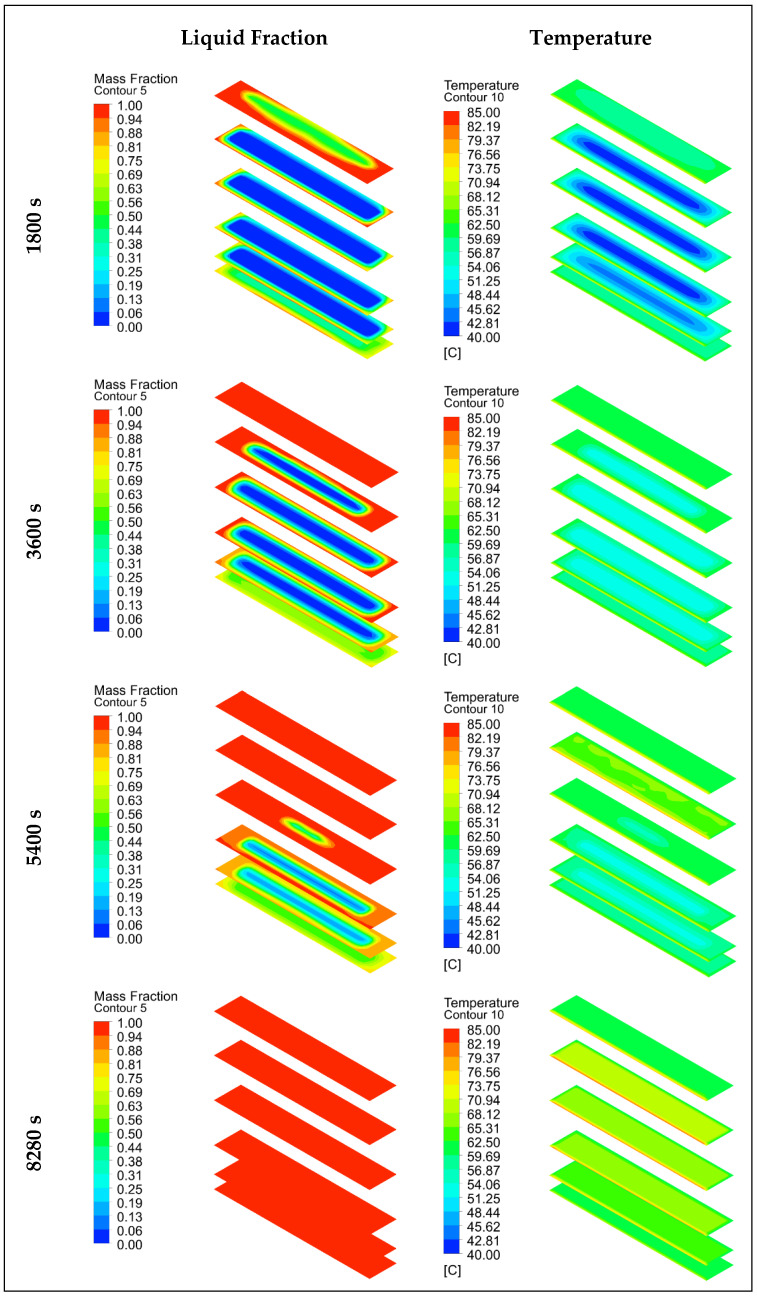
Temperature and liquid fraction contours of P1 during the melting (charging) process.

**Figure 12:**
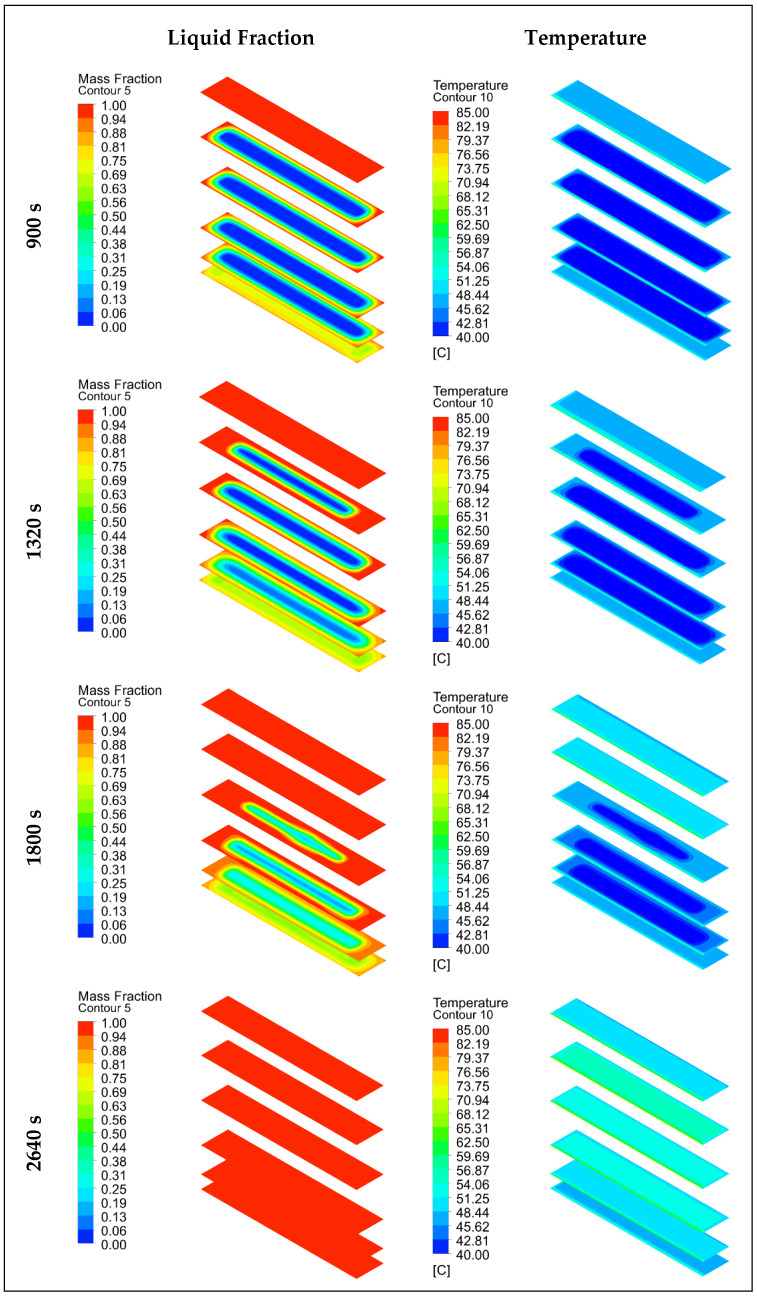
Temperature and liquid fraction contours of P2 during the melting (charging) process.

In this section, the results of the numerical analysis of the designed TESU are presented. The experimental study used three different HTF flow rates (0.0333 lt/s, 0.0666 lt/s, and 0.1 lt/s) and three different temperatures (60 °C, 70 °C, and 80 °C). Numerical analyses were performed according to these operating conditions, and the results are presented for P1, P2, EP1, and EP2 at a temperature of 80 °C and a flow rate of 0.0333 lt/s, which were selected as the reference values. In the numerical modeling for P1 and EP1, the complete melting and solidification temperatures were taken as 60.4 °C and 52.3 °C. Figure 11 and Figure 12 show the time-dependent temperature contours and liquid fraction for the 3D model’s melting (charging) process, as shown in Figure 5. The numerical model was assigned six planes on the XZ-axis. The topmost plane was named plane 1, and the bottom plane was called plane 6. The distance between all the planes except for plane 5 was 5.75 cm. The distance between planes 5 and 6 was 2 cm, and that between planes 5 and 4 was 3.75 cm. The thermocouples shown in Figure 3a are in plane 5 in the numerical model. As can be seen from the figures, the liquid fraction varied between 0 and 1, with blue representing solid paraffin and red representing liquid molten paraffin. The colors green and yellow, which fall between red and blue, stand for the mushy zone, the transitional region between solid and liquid.

The TESU was designed with a copper heat exchanger integrated on one surface. The heat from the electric heater in the water tank was first transferred to the copper heat exchanger and then to the paraffin in the TESU. In this manner, heat transfer initially occurred through convection from the surface of the TESU to the walls of the heat exchanger, as well as through conduction from the walls of the heat exchanger to the surface of the TESU, and then to the paraffin. The paraffin gradually began to melt as the temperature approached its melting point. After that, heat was transferred via convection between the TESU and the melted paraffin. The melted paraffin gradually rose towards the upper region of the TESU due to its low density.

Figure 11 shows the temperature and liquid fraction contours of P1, and Figure 12 shows the temperature and liquid fraction contours of P2 during the melting process. As seen from both figures, the melting rate of paraffin was higher on the front surface where the heat exchanger was integrated. However, since the TESU was constructed of very thermally conductive copper, the heat from a single surface was quickly transferred to all the other surfaces, and the paraffin melted faster when it contacted the walls of the TESU. In this way, the melting profile in the XZ-axis was from the surfaces to the middle parts of the TESU. In the numerical analysis, when the melting profile in the vertical direction of the XY-axis and YZ-axis was examined, it was observed that most of the paraffin that melted during charging was on the ceiling of the unit, and it was determined that natural convection had an essential effect on the charging process and increased the heat transfer. As seen in Figure 11, at 5400 s, and 12, at 1800 s, the paraffin melted last in the lower plane. Due to the low heat transfer coefficient of paraffin, the melting process was prolonged because the heat was transferred to the solid paraffin in places far from the walls of the TESU.

**Figure 13:**
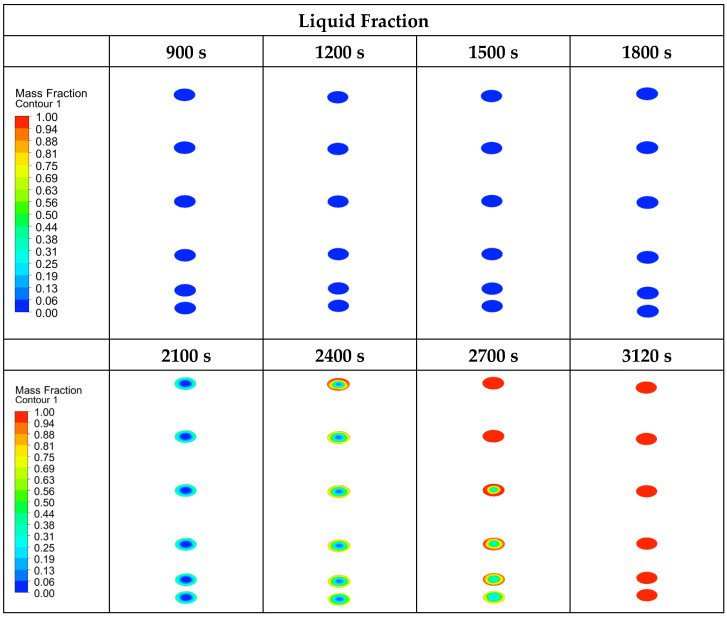

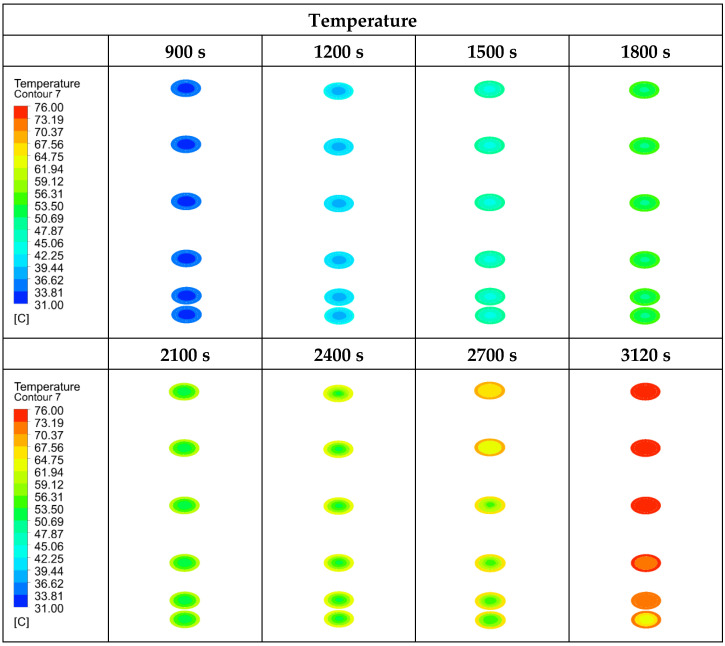
Liquid fraction and temperature contours of EP1 during the melting (charging) process.

**Figure 14:**
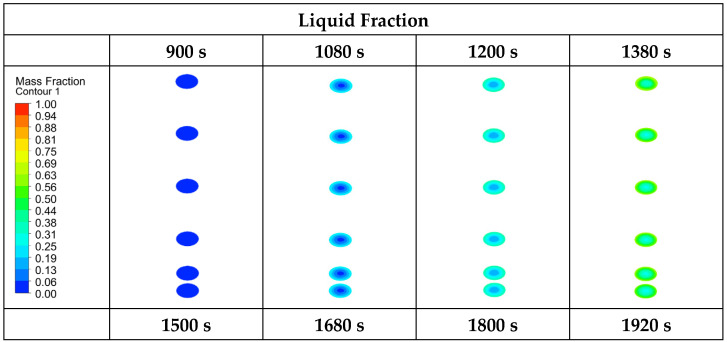

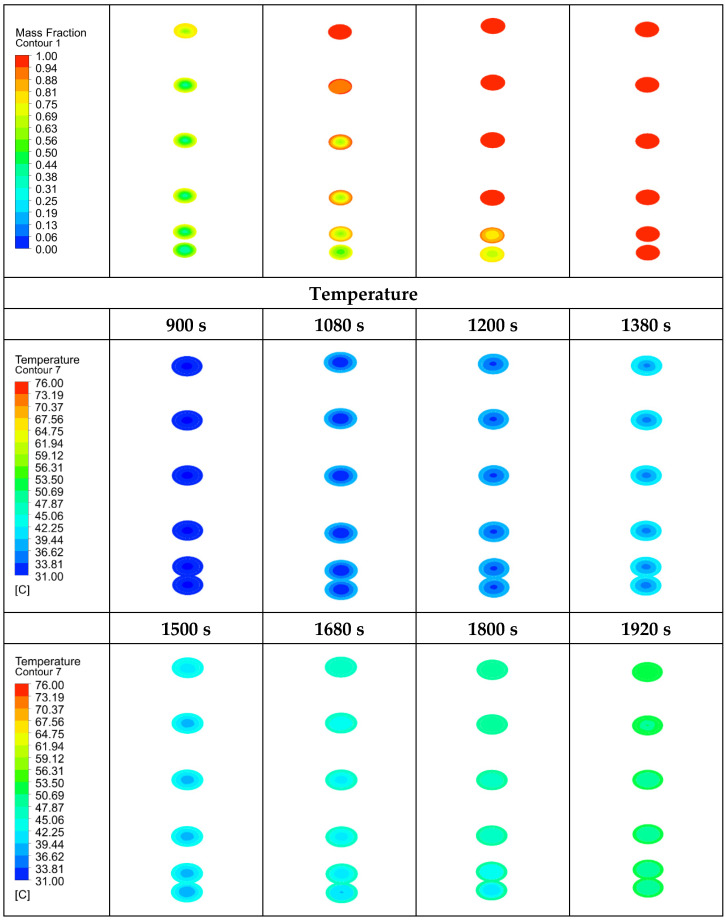
Liquid fraction and temperature contours of EP2 during the melting (charging) process.

In the numerical model for P2 and EP2, the complete melting and solidification temperatures were taken as 46.1 °C and 31.7 °C. Figure 13 and Figure 14 show the time-dependent temperature and liquid fraction contours for the melting process of the 3D model given in Figure 8b. Six planes were assigned on the XZ-axis in the numerical model of the encapsulated paraffins. Figure 13 shows the liquid fraction and temperature contours of both the paraffin and the capsule (outer side of capsule, inner side of paraffin) during the melting process of encapsulated paraffin-1. As can be seen, the liquid fraction of EP1 was 0 in the first 1800 s. This was due to the wall thickness of the aluminum capsule. The heat flux defined on the outer surface of the capsule traveled to the inner surface of the capsule by conduction. When the inner surface of the capsule reached a sufficient temperature, melting started. The paraffin melted faster in the places where it contacted the capsule walls. In this way, the melting profile in the XZ-axis was from the capsule surfaces to the middle parts. Due to the effect of natural convection, melting in the vertical direction took place from the upper plane to the lower plane. Compared to Figure 11, unencapsulated paraffin-1 melted in 8280 s, while encapsulated paraffin-1 melted in 3120 s. Therefore, the macrocapsule contributed 62.32% to the heat recovery in this storage system during the charging process.

Figure 14 shows the liquid fraction and temperature contours of both the paraffin and the capsule (outer side of capsule, inner side of paraffin) during the melting process of encapsulated paraffin-2. Compared to EP1, it melted in a shorter time, as it had a lower melting point, and the liquid fraction in the first 900 s was 0 due to the wall thickness of the aluminum capsule. The heat flux was conducted from the outer surface of the capsule and reached the inner surface. When the temperature on the inner surface reached a sufficient level, the paraffin started to melt. Compared to Figure 12, unencapsulated paraffin-2 melted in 2640 s, while encapsulated paraffin-2 melted in 1920 s. This paraffin with a low thermal conductivity provided an increase of 27.27% in the efficiency of the LHS system during charging with the macroencapsulated PCM.

### 4.3. Experimental Analysis Results

**Figure 15:**
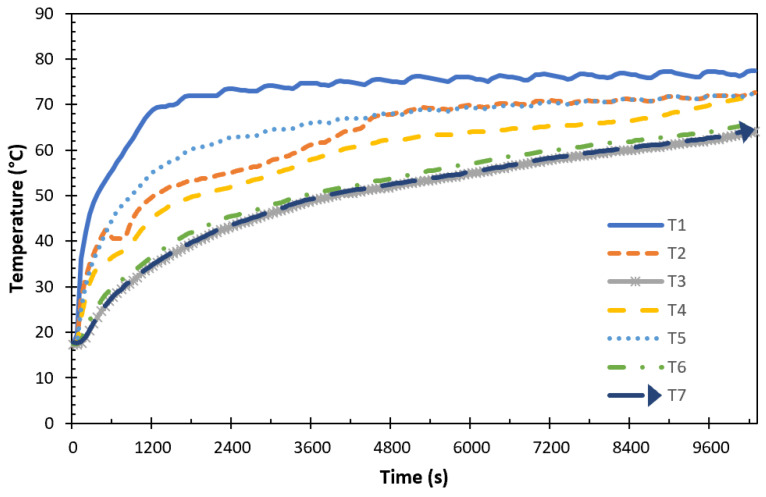
Time-dependent temperature variations on the same plane during melting (P1, $T_{HTF}$ = 80 °C ve ${\dot{Q}}_{HTF}$ = 0.0333 lt/s).

The experimental findings and discussions about the paraffin-1, paraffin-2, encapsulated paraffin-1, and encapsulated paraffin-2 in the TESU are presented in this section. To examine the temperature profiles in the storage unit and to draw the graphs, P1 was taken as a reference PCM, and a temperature of 80 °C (temperature in the water tank) and a flow rate of 0.0333 lt/s were selected as references for the HTF. Figure 15 displays the temperature profiles that were acquired during charging from several thermocouples in P1.

**Figure 16:**
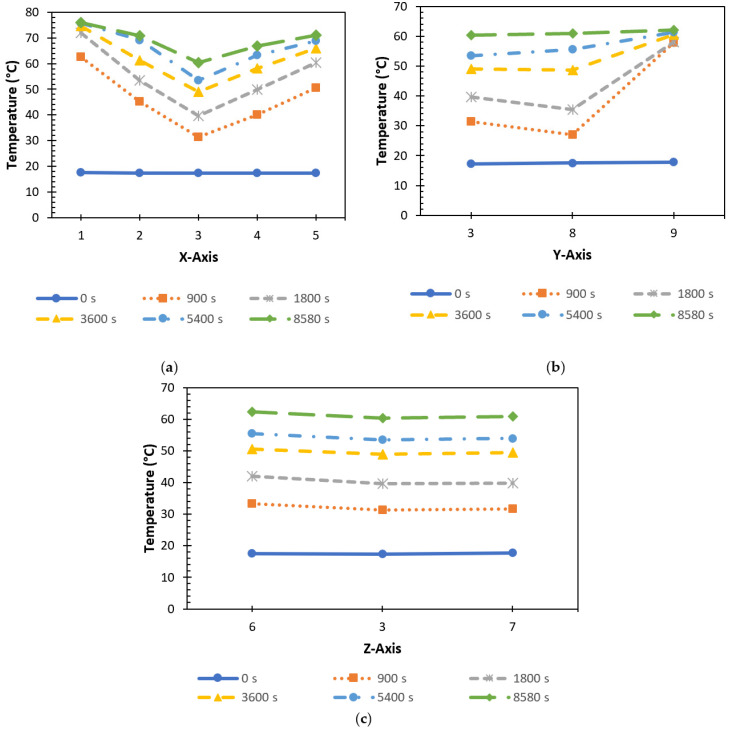
Time-dependent axial and radial temperature variations for (**a**) the X-axis, (**b**) the Y-axis, and (**c**) the Z-axis during melting (P1, $T_{HTF}$ = 80 °C ve ${\dot{Q}}_{ITA}$ = 0.0333 lt/s).

The seven thermocouples shown in Figure 15 were located in the plane 5 position mentioned in the numerical analysis results, where the melting process was completed last. Since the thermostat constantly fluctuated between the on and off state to reach the desired operating temperature, a stable HTF temperature could not be practically maintained while the system was running. For this reason, as can be seen in the figure, T1 was located closest to the HTF inlet and was affected by this temperature fluctuation throughout the melting process. During the initial heating phases, a consistent temperature increase was noted everywhere, signifying heat conduction. As the temperature rose, this was followed by a steep slope transition that indicated the PCM’s storage of latent heat. The axial and radial temperature profiles of all the thermocouples placed in the TESU during the melting process are shown in Figure 16.

The charging process in the TESU was affected by heat transfer in both the radial and axial directions. In Figure 16a, the temperature locations T1, T2, T3, T4, and T5 are displayed at various time intervals to calculate the axial variation. As can be seen, initially at t = 0 s, the temperatures at all the locations were in equilibrium. The heat transfer fluctuation in the axial direction was anticipated to diminish, since the heat source, the HTF, ran the whole length of the thermal energy storage module. However, since the storage unit was composed of copper material and copper has a high heat conduction coefficient, the heat from the only surface contacted by the heat exchanger was transferred to all the other surfaces very quickly, and the paraffin melted faster where it contacted the walls of the TESU. Heat transfer from the HTF to the PCM raised its temperature. Because of the large temperature differential between the PCM and the HTF, the heat transfer rate was initially higher near the intake portion. There was a drop in the heat transfer rate as time went on because the temperature differential between the PCM and the HTF decreased along the length up to temperature location T3. After T3, the temperature increased at T4 and T5 due to the effect of heat from the other surfaces. As a result, the melting process in the axial direction took place at temperature positions T1, T5, T2, T4, and T3. In addition, as can be seen from the graph, the progression of time from t = 900 s to t = 8580 s resulted in the temperatures at all the axial positions approaching each other and reaching a more stable state.

As seen in Figure 16b, the evolution of the temperatures at locations T3, T8, and T9 were plotted and examined at various time intervals in order to determine the change in the radial direction. At the beginning, at t = 0 s, the temperatures were the same everywhere. Then, natural convection began to occur when the PCM melted. The heat rose toward the top of the PCM-filled melting region as a result of natural convection, and the molten liquid PCM remained in the higher regions due to its lower density. The rate of melting was very high at first and then gradually decreased. Throughout the charging process, melting in the upper zone (T9) occurred faster than in the middle and lower zones. When comparing the middle (T8) and lower (T3) regions, the temperature of the lower area was higher than that of the middle region due to the effect of conduction heat transfer from the bottom of the TESU to the paraffin until t = 1800 s. At t = 3600 s, the temperatures at T3 and T8 were almost identical. In the remaining time, as natural convection became more effective, the temperature increase in the middle region became higher than in the lower area. At t = 8580 s, it was observed that the temperatures at all radial positions approached each other and reached a steady state. As a result, melting in the radial direction took place at temperature positions T9, T8, and T3.

To determine the variation in the Z direction, the temperature locations T6, T3, and T7 were displayed at various time intervals, as shown in Figure 16c. Initially, at t = 0 s, the temperatures at all the locations were equal. At the beginning of the charging process, from t = 0 s to t = 900 s, the heat transfer rate was high because of the high-temperature difference between the PCM and the HTF. When the temperature profile in the Z direction during the charging process was examined, it was observed that the temperature at T6 was higher due to the effect of heat transfer from the front surface of the storage unit to the paraffin, where the heat exchanger was in contact. This was followed by the temperature at T7 and T3, with these locations being the furthest from the walls of the storage unit. There were no significant differences between the temperatures due to the small distance in the Z-axis.

**Figure 17:**
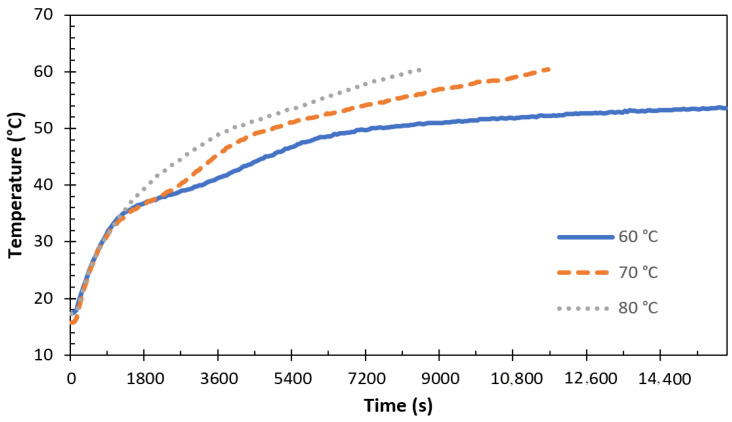
Effect of HTF temperature on the charging performance (P1 ve ${\dot{Q}}_{ITA}$ = 0.0333 lt/s).

**Figure 18:**
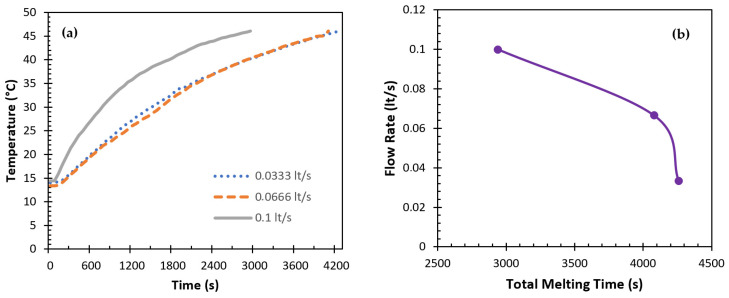
Effect of the HTF flow rate on the charging performance of the TESU (P2 ve $T_{HTF}$ = 60 °C): (**a**) temperature–time and (**b**) flow–time.

The effect of the HTF inlet conditions on charging is analyzed in Figure 17 and Figure 18. Figure 17 shows the temperature change for P1 inside the storage unit by varying the HTF inlet temperature.

In this experiment, as shown in Figure 17, the flow rate was fixed at 0.0333 l/s. The temperature of thermocouple T3, at the last position of the melting process, was taken into account. The differential between the HTF and the PCM’s starting temperature rose when the HTF’s intake temperature increased. The TESU’s natural convection and conduction heat transfer rates were accelerated. By increasing the HTF temperature from 60 °C to 70 °C, a significant increase in the T3 temperature was observed. However, a precise figure regarding the PCM melting time cannot be given because the HTF temperature of 60 °C was insufficient for the complete melting of P1. P1 reached its melting point at 13,140 s. However, the experiment was terminated because there was not even a 1 °C increase in its temperature in the following 2820 s. To investigate the effect of the HTF inlet temperature on the melting time of paraffin, the HTF inlet temperature was increased from 70 °C to 80 °C, resulting in a 26.3% decrease in the charging time from 11,640 s to 8580 s. The rate and quantity of heat stored were significantly increased by raising the differential between the starting PCM temperature and the intake HTF temperature. Figure 18 shows the temperature change for P2 and the change in the total melting time by varying the HTF flow rate.

To determine how the intake HTF flow rate affected the charging efficiency, the flow rate was adjusted from 0.0333 to 0.1 l/s; the initial PCM temperature was fixed at 13–14 °C; and the HTF inlet temperature was fixed at 60 °C. For paraffin, the results obtained for P2 were used, since P1, which was selected as the reference PCM, could not melt completely at an HTF temperature of 60 °C. Figure 18a demonstrates that thermocouple T3’s temperature increased with an increasing water flow rate. However, significant temperature differences were only observed when a certain level was reached. On the other hand, increasing the HTF flow rate decreased the total melting time (Figure 18b). When the flow rate was increased from 0.0333 lt/s to 0.0666 lt/s, the total melting time decreased to 4.2%, and when the flow rate was increased from 0.0666 lt/s to 0.1 lt/s, it decreased to 27.9%. The observed decrease in the overall melting time at increasingly higher flow rates might be attributable to this thermal behavior, which could also be linked to increased heat transfer. In addition, all flow rates were in the laminar regime.

It is not possible to directly compare the impact of raising the HTF inlet temperature or flow rate. The results demonstrate that the inlet HTF temperature has a considerable impact on the heat transfer rate, since both can vary significantly. On the other hand, it is evident that raising the intake flow rate over a threshold value causes the heat transfer rate to increase significantly.

**Figure 19:**
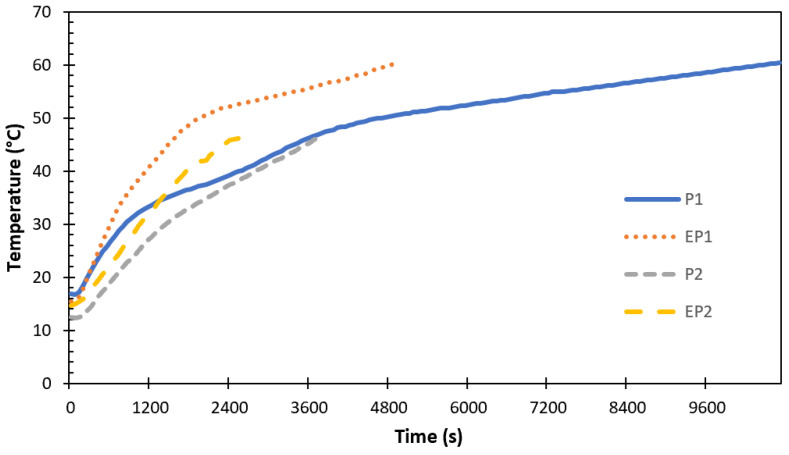
Effect of PCM encapsulation on the charging performance of the TESU ($T_{HTF}$ = 70 °C ${\dot{Q}}_{ITA}$ = 0.0666 lt/s).

After a detailed study of the effect of the HTF inlet conditions on the charging of the storage unit, the impact of PCM encapsulation on the charging performance of the TESU was investigated and the results are presented in Figure 19.

The experiments were carried out using different paraffins under all conditions, at different temperatures, and at various flow rates of the HTF. Experiments were also carried out by encapsulation under the same conditions. As shown in Figure 19, the temperature–time graph was plotted for two different paraffins with and without encapsulation under certain conditions for comparison, with the HTF inlet temperature fixed at 70 °C and the flow rate fixed at 0.0666 l/s. Due to experimental limitations, there was a difference (~3–4 °C) between the initial temperatures of paraffin due to fluctuations in the ambient temperature. On the other hand, the behavior of heat transfer was obviously shown by the temperature variation over time, and there was an increase in all these temperatures. The total melting time decreased to 53.9% with the encapsulation of paraffin-1, which had a low thermal conductivity and a high specific heat, and 32.2% with the encapsulation of paraffin-2. This was related to an increased heat transfer surface area and thermal conductivity with aluminum capsules. The results show that the encapsulation process improves the thermal properties of PCMs by increasing the heat transfer area, resulting in a faster heat transfer during charging processes between the PCM and its surroundings.

**Figure 20:**
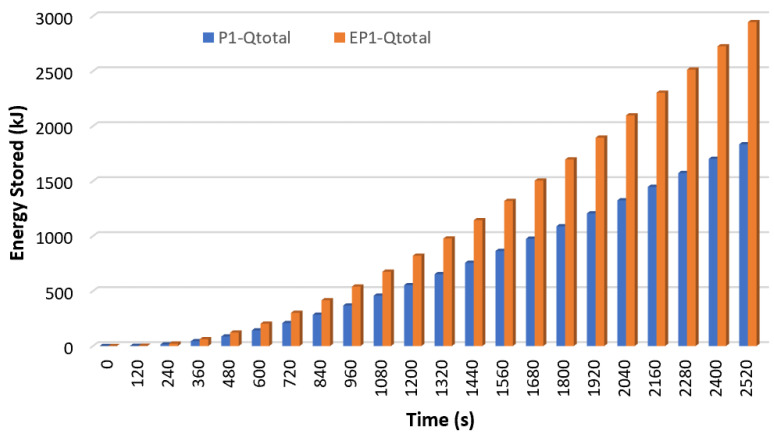
The total energy stored at the same time for P1 and EP1 ($T_{HTF}$ = 70 °C ve ${\dot{Q}}_{ITA}$ = 0.0666 lt/s).

**Figure 21:**
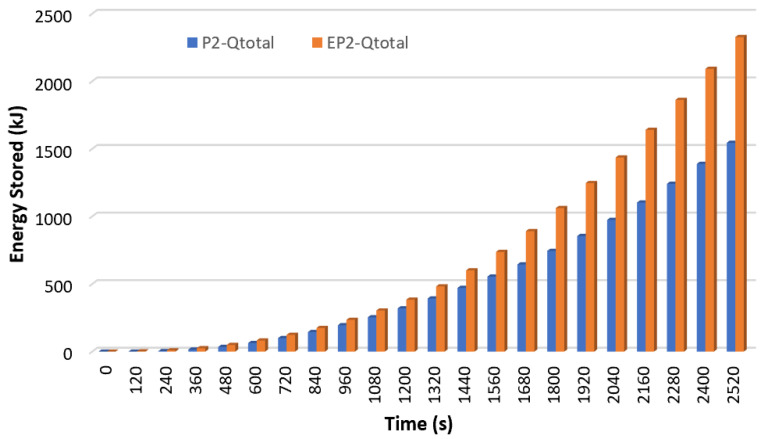
The total energy stored at the same time for P2 and EP2 ($T_{HTF}$ = 70 °C and ${\dot{Q}}_{ITA}\;$= 0.0666 lt/s).

Figure 20 shows the total energy stored at the same time for P1 and EP1, and Figure 21 shows the total energy stored at the same time for P2 and EP2.

The sensible heat and latent heat stored by P1 and EP1 over time at 2520 s were summed, and the total energy stored simultaneously is shown in Figure 20. As seen from here, encapsulated paraffin showed the best thermal performance. At the end of 2520 s, P1 stored 1834.9 kJ of energy while EP1 stored 2945.8 kJ. With the encapsulation of paraffin-1, the energy stored simultaneously increased by 60.5%, and thus, the charging rate increased. Similarly, when the total energy stored for P2 and EP2 was analyzed, as shown in Figure 21, it was observed that the encapsulated paraffin stored more energy. At the end of 2520 s, P2 stored 1544.8 kJ of energy while EP2 stored 2327.6 kJ. It was understood from this that the energy stored simultaneously increased by 50.7% with the encapsulation of paraffin-2. Since the two figures were drawn at the same HTF temperature and flow rate ($T_{HTF}$ = 70 °C and ${\dot{Q}}_{ITA}$ = 0.0666 lt/s) and for the same length of time (2520 s), it was possible to compare the energy stored by the two different paraffins. At the same time, P1 stored 15.8% more energy than P2. This can be explained by the fact that P2 had a lower latent heat and specific heat (see Table 1).

## 5. Conclusions

In this study, a thermal energy storage unit was designed using cylindrical-shell aluminum macrocapsules with a high thermal conductivity and a high mechanical strength to minimize the disadvantages of paraffin, such as its low thermal conductivity, its limited operating temperature range, and leakage and stabilization problems. The thermal behavior of paraffin in the TESU was investigated both experimentally and numerically. The numerical analysis was carried out using the Ansys 18.2-Fluent software. The thermal energy storage performances of two different paraffins, encapsulated and unencapsulated, whose properties were determined by differential scanning calorimetry, were compared. The effect of the HTF inlet conditions on the charging (melting) performance was investigated. When the numerical and experimental analysis results were analyzed, the following conclusions were reached:

- It was found that the inlet temperature of the HTF significantly affected the heat transfer rate. It was observed that an increase in the HTF temperature decreased the charging time by 26.3%.
- Increasing the inlet flow rate above a threshold value increased the heat transfer rate by 27.9%.
- Since the thermal conductivity and heat transfer surface increased with the encapsulation of paraffin, a significant decrease in the total melting time was observed. Encapsulation proved to be one of the most promising methods of increasing heat transfer.
- The total energy stored simultaneously increased by 60.5% due to the encapsulation of paraffin-1 and 50.7% due to the encapsulation of paraffin-2.
- Aluminum capsules with a high thermal conductivity and mechanical strength showed excellent potential as an inexpensive encapsulation material.
- The encapsulation technique kept the liquid phase and solid phase of the paraffin in a specific volume and avoided the risk of leakage from the TESU.
- Heat transfer was found to occur predominantly through natural convection during the charging process. For this reason, melting in the radial direction during the charging process occurred in the upper, middle, and lower regions.
- The model was validated, and it was found that the results of the numerical analysis and the experimental results agreed.

## Figures and Tables

**Table 1:** Thermophysical properties of paraffin and copper.

Symbols	Explanations	Values Paraffin-1 (P1)	Values Paraffin-2 (P2)	Values Copper
T_m_	Melting temperature	52.9 °C *	32.2 °C *	N.A.
T_p_	Peak temperature	60.4 °C *	46.1 °C *	N.A.
L_s_	Latent heat (solid)	128 kJ/kg *	6.91 kJ/kg *	N.A.
L_l_	Latent heat (liquid)	132 kJ/kg *	33.7 kJ/kg *	N.A.
C_p_	Specific heat	2.41 kJ/kg.K *	1.61 kJ/kg.K *	0.380 kJ/kg.K
ρ_s_	Density (solid)	850 kg/m^3^ ***	957 kg/m^3^ **	8920 kg/m^3^
ρ_l_	Density (liquid)	750 kg/m^3^ ***	895 kg/m^3^ **	N.A.
k	Thermal conductivity	0.2 W/m.K ***	0.2 W/m.K ***	398 W/m.K

* Received from DSC device. ** Calculated. *** Obtained from literature [12].

## Data Availability

The data are contained within the article.
